# RNA interfering machinery: new insight into the Dicer-2 complexes in the dsRNA-processing cycle

**DOI:** 10.1038/s41392-022-01272-9

**Published:** 2023-01-02

**Authors:** Xiaocui Wei, Junxia Li, Yang Li

**Affiliations:** 1 CAS Key Laboratory of Animal Ecology and Conservation Biology, Institute of Zoology, Chinese Academy of Sciences, Beijing, China; 2 School of Life Sciences, Hebei University, Baoding, China; 3 University of Chinese Academy of Sciences, Beijing, China

**Keywords:** Structural biology, RNAi

Two emerging studies published in *Nature* back to back reported the cryo-electron microscopy (cryo-EM) structures of Dicer complexes in the double-stranded RNA (dsRNA)-processing cycle, providing detailed mechanistic insights into the role of Dicer-2 in dsRNA-siRNA processing (dsRNA binding, translocation, dicing, strand selection, and siRNA loading).^1,2^

siRNAs are a crucial component for RNA interference (RNAi), a well-known gene expression regulation mechanism in various eukaryotes. Dicer, an RNase III enzyme, has a central role in producing siRNAs. A canonical Dicer protein contains a helicase domain, a PAZ domain, a domain of unknown function (DUF283), two RNase III domains, and a dsRNA binding domain.^3^ Dicer plays a vital role in cellular homeostasis and fighting virus infections.^4^ The *Drosophila* encodes two Dicers annotated as Dicer-1 and Dicer-2. Dicer-1 interacts with the PB isoform of dsRBP Loquacious (Loqs-PB) to process microRNAs (miRNAs) precursors. By contrast, ATP-dependent Dicer-2 produces siRNA duplexes by cleaving long dsRNAs with the help of its cofactor Loquacious-PD (Loqs-PD). These siRNA duplexes are loaded into Argonaute2 (Ago2) protein with the aid of another partner protein, R2D2.^3^ It’s known that Dicer-2 mediates the detection of virus-derived specific dsRNAs and the processing of these dsRNAs into siRNAs in antiviral RNAi responses.^4^ So far, several biochemical and structural studies on Dicer proteins have been reported. However, the dynamic molecular mechanism of how the Dicer-2 cleaves dsRNAs to generate siRNAs and how siRNAs are loaded into Ago2 in a defined orientation have not been fully elucidated.

**Fig. 1:**
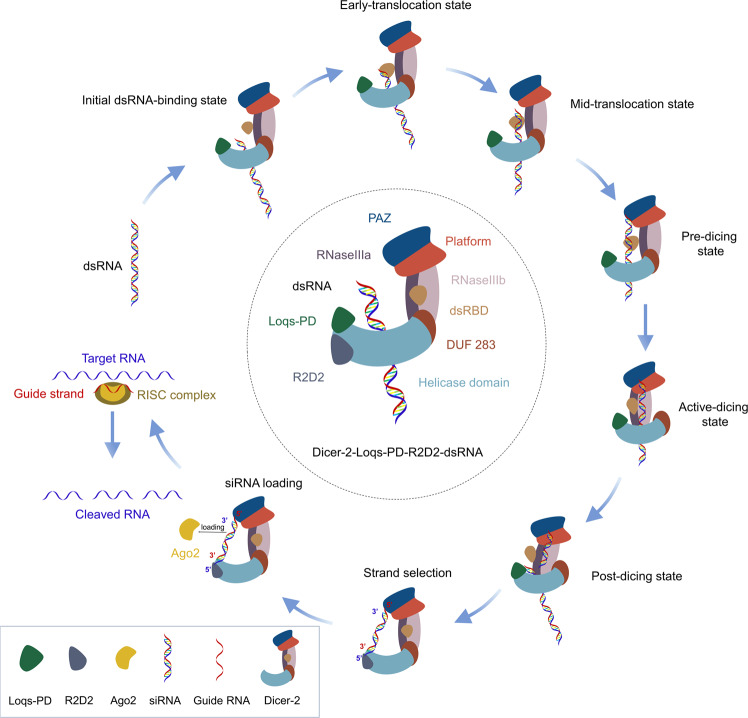
Schematic diagram demonstrating the full cycle of Dicer-2 complexes in dsRNA processing. Starting from the recognition of dsRNA on the left, the step by step of dsRNA processing by Dicer in a clockwise direction. In the middle is a detailed view of the Dicer-2-Loqs-PD-R2D2-dsRNA structure

One of the two studies, from the teams of Jinbiao Ma, Fudan University, and Hongwei Wang, Tsinghua University, revealed the mechanism for the cycle of ATP-dependent dsRNA processing by Dicer-2-Loqs-PD.^1^ Using wild-type (WT) Dicer-2 and its mutant Dicer-2, with different combinations of assembly components, they carried out the structural analysis of the Dicer-2-Loqs-PD in the apo state and in multiple states. They successfully captured six structures including the apo state of Dicer-2-Loqs-PD complex, the complex of Dicer-2-dsRNA-Loqs-PD in the initial binding pattern without ATP, the dsRNA-loading state and the active dicing state with ATP, and the WT Dicer-2-Loqs-PD complex in post-dicing state (Fig. 1). Through continuous structural analysis of the Dicer-2-dsRNA-Loqs-PD complex from the initial binding pattern to the translocation state and then to the active dicing state, they showed that Loqs-PD has only an assisting function for the Dicer-2. It is worth mentioning that a previously unstudied structure appears among these complexes. The Dicer-2-dsRNA-Loqs-PD complex formed a special ring-like structure in the initial binding state, which may indicate some functions of the complex that we do not yet understand. Further, they demonstrated that dsRNA threaded through the helicase domain towards the catalytic center of Dicer-2-Loqs-PD complex during the translocation and cleavage of dsRNA. In addition, they pointed out the central role of the DUF283 domain in the conformation changes of the Dicer-2-dsRNA-Loqs-PD, recognizing dsRNAs by interacting with the helicase domain and blocking non-specific cleavage of dsRNAs by interacting with the RIIIDa/b region. Overall, this study successfully solved the structure of the Dicer-2-Loqs-PD complex by cryo-EM, providing the details of the full cycle of ATP-dependent dsRNAs processing, including dsRNAs binding, translocation, and dicing.^1^

Another study came from the group of Osamu Nureki, Hiroshi Nishimasu, and Yukihide Tomari, The University of Tokyo.^2^ They reported the structure of the Dicer-2-R2D2 heterodimer and the complexes in the RNA binding state. Notably, this study first showed the structures of the Dicer-2-R2D2 complex bound to RNAs represent dsRNAs recognition and cleavage, siRNAs strand selection, and loading state, respectively (Fig. 1). It is worth noting that by analyzing the spatial structure of the Dicer-2-R2D2 complex, they demonstrated an α-helical region inserted in the RIIIa domain of the Dicer protein. By comparing the structure of Dicer-2-R2D2 heterodimer with Dicer-2-siRNA-R2D2, they found that the structure of the R2D2 protein changes when it bound to the siRNA. Furthermore, through structural comparison between fly Dicer-2-R2D2 heterodimer and human Dicer-pre-miRNA-TRBP, they pointed out that there is a spatial clash when R2D2 binds to miRNA precursors, explaining why R2D2 inhibits pre-miRNAs processing by Dicer-2 protein. They also revealed that R2D2 binds to siRNAs in a sequence-independent manner. More importantly, by analysis of those structures, they illustrated the mechanism that the Dicer-2-R2D2 complex asymmetrically recognized the different siRNA duplex with higher binding capacity and thus determined which strand of the siRNA duplex was selected by Ago2.^2^ These results provide a better understanding of how Dicer2-R2D2 functions when it comes to recognizing dsRNA and selecting siRNA for pre-loading.

Altogether, these two works are carried out from the dsRNA loading and cleavage, and delivery of siRNA by Dicer-2 and cofactors, respectively, jointly elaborating the full cycle of Dicer-2 protein processing dsRNA into siRNA (Fig. 1). To further clarify the molecular principles of the RISC based on these studies, future work should focus on elucidating the structure of the Dicer-2-siRNA-R2D2-Ago2 complex. The fly Dicer-2 is essential for antiviral activity in invertebrates.^4^ In many organisms, including humans, a single-Dicer generates both siRNA and miRNA by association with one or more cofactors. And unlike fly Dicer-2, RNA processing for human Dicer needs ATP.^3^ Previous studies indicated that human Dicer processed long dsRNAs less efficiently than miRNA precursors in vitro. However, recent studies have demonstrated the production of abundant virus-derived small-interfering RNAs during the infection of undifferentiated and differentiated mammalian cells with distinct positive- or negative-strand RNA viruses, suggesting efficient Dicer processing of virus-derived dsRNAs in mammals.^5^ How the single human Dicer recognizes and processes dsRNA into siRNA and what cellular cofactors of Dicer mediate this process remains to be resolved.
